# Macrophage-Derived MMP-9 and MMP-2 are Closely Related to the Rupture of the Fibrous Capsule of Hepatocellular Carcinoma Leading to Tumor Invasion

**DOI:** 10.1186/s12575-023-00196-0

**Published:** 2023-03-14

**Authors:** Quanwei Cui, Xuben Wang, Yongwei Zhang, Yiqing Shen, Yeben Qian

**Affiliations:** 1 Department of General Surgery, The First Affiliated Hospital of Anhui Medical University, 218 JiXi Road, Hefei, Anhui 230022 China; 2 Division of Molecular Medicine, Hefei National Laboratory for Physical Sciences at Microscale, The CAS Key Laboratory of Innate Immunity and Chronic Disease, School of Life Sciences, University of Science and Technology of China, Hefei, Anhui China; 3 Department of General Surgery, Anqing First People’s Hospital Affiliated to Anhui Medical University, Anqing, Anhui 246004 China

**Keywords:** Hepatocellular carcinoma (HCC), MMP-2, MMP-9, Fibrous capsule

## Abstract

**Background:**

Hepatocellular carcinoma (HCC) is an aggressive tumor with a poor clinical prognosis. Rupture of the fibrous capsule (FC) is a very important clinical phenomenon in the invasion and metastasis of HCC. FC is mainly composed of type I collagen (COL1A1). However, it is not clear what caused the FC rupture. In this study, we aimed to determine whether the rupture of FC in HCC patients was related to macrophage-derived MMP-9 and MMP-2, and their clinical diagnostic value for FC rupture.

**Results:**

By performing immunohistochemical and immunofluorescence staining of ruptured FC and intact FC, the results showed that the ruptured area of FC aggregated a large number of macrophages with MMP-9 and MMP-2. Western blot analysis and Quantitative real-time PCR were used to assess the expression of MMP-9 and MMP-2 in the ruptured and relatively intact area of FC in ruptured FC patients, and the results revealed a significantly different expression of MMP-9 and MMP-2. ELISA experiments show that we could discriminate effectively between ruptured FC and intact FC by MMP-9 and MMP-2.

**Conclusions:**

Taken together, macrophage-derived MMP-9 and MMP-2 were closely related to the rupture of the FC of HCC and subsequently led to the migration and invasion of the tumor cells through the ruptured area of FC to the para cancer. It is suggested that when performing surgical resection, it is necessary to expand the range of tumor resection for patients with ruptured FC and hence reduce the possibility of recurrence and metastasis in HCC patients.

## Highlights

• HCC capsule broken leading to tumor invasion

• Macrophage-derived MMP-9/MMP-2 were closely related to the rupture of the FC of HCC

• MMP-9/MMP-2 was able to discriminate effectively between ruptured FC and intact FC

**Graphical Abstract:**

## Graphical abstract

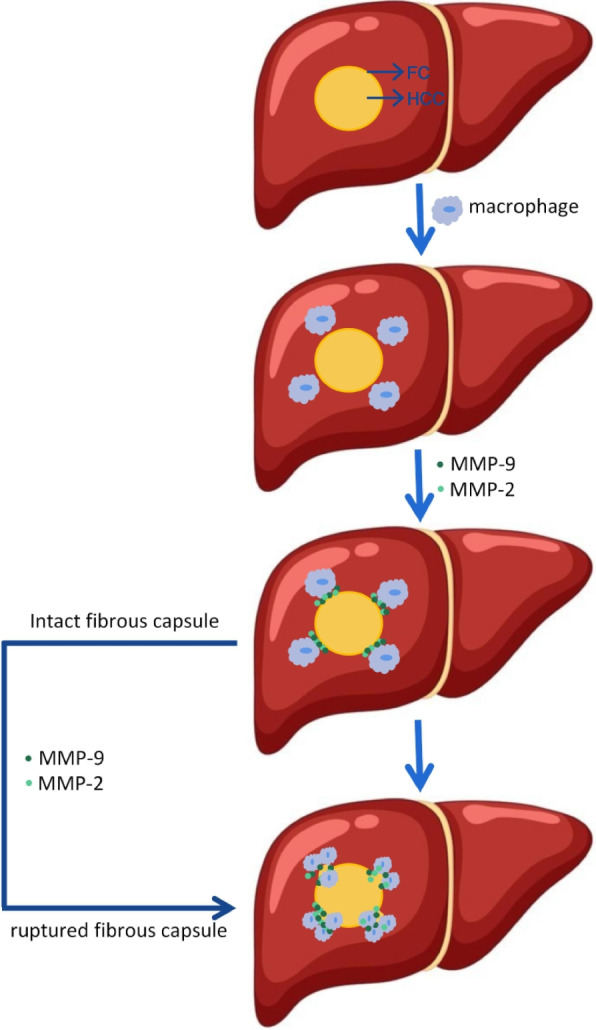

## Introduction

Hepatocellular carcinoma (HCC), which originates from the hepatocytes and accounts for 80%-90% of primary liver cancer [1–3] is the fifth most common cancer worldwide. It is also the third leading cause of cancer-related death and the incidence is rapidly growing [4–7]. In recent years, with the continuous development of novel diagnosis and treatment methods, the overall survival rate in HCC patients has significantly improved but is still unacceptable [8, 9]. HCC is characterized by high migration as well as invasion abilities and can display high metastatic potential [10–13] and the occurrence of intrahepatic or systemic metastases is a principal cause underlying its high mortality and poor prognosis [14, 15]. It is worth mentioning that the presence of FC around HCC plays a critical role in limiting the migration and invasion of tumor cells and has been closely related to tumor progression and long-term survival. In addition, the presence of ruptured or not FC has been reported to serve as a prognostic factor for HCC patients [16], and hence it is necessary to study mechanisms underlying the destruction of HCC fibrous capsule.

Although the potential mechanisms of FC formation and destruction are not clear, several prior studies have reported that FC is mainly composed of type I collagen [17, 18]. Type I collagen is mainly composed of type Iα1 collagen (COL1A1) [19, 20], which is the major component of the extracellular matrix (ECM) [17]. Moreover, in the presence of liver injury, hepatic stellate cells (HSCs) can be activated from a quiescent state to highly proliferative myofibroblast-like cells expressing α-SMA, thus producing a considerable amount of ECM responsible for the formation of tumor FC, which can subsequently impact the migration and invasion of the tumor cells [17, 18]. The FC formed by type I and type III collagen is thought to be a barrier that can effectively restrict the spread of HCC cells [18, 21, 22] and can prevent the invasion of HCC into the paracancerous liver tissues. Clinically, we have observed that some patients have extremely irregular tumor shapes, such as spear-shaped tumors with non-intact FC. Due to the lack of an intact FC, these patients have a poor post-operative prognosis and are prone to recurrence in the short term; In contrast, some patients with HCC have a spherical shape due to the existence of an intact FC on the surface of the tumor, the tumor is extremely intact and surrounded by an intact FC, so the boundary between the cancerous tissue and the para-cancerous liver tissue is quite clear, and these patients have a good post-operative prognosis and are not prone to recurrence. Therefore, FC rupture is a vital clinical phenomenon in HCC invasive metastasis and tumor recurrence.

Matrix metalloproteinases (MMPs) are a family of zinc-containing enzymes that can degrade ECM and promote tumor invasion as well as metastasis [23–27]. They are commonly found in malignant tumors and their peri-malignant tissues as compared to the normal and benign tissues, with the highest expression occurring in the actively invasive area of the tumor-mesenchymal junction [28]. MMP-9 and MMP-2 (also called gelatinases) are two of the most extensively studied members of the MMPs family [29], and over-expression of MMP-9 and MMP-2 in HCC can contribute to a higher Tumor-Node-Metastasis (TNM) stage by promoting the tumor cells to undergo metastasis as well as invasion and can also cause poor differentiation and overall poor prognosis. MMP-9 and MMP-2 are not produced specifically by HCC cells, but by myofibroblasts (activated HSCs) [30, 31]. Accumulating evidence has demonstrated that there are two major macrophage types in the tumor microenvironment: M1 and M2. M1 macrophages possess the tumor-killing activity and are characterized by the production of tumor necrosis factor (TNF) and IL-6. In contrast, M2 macrophages can act as tumor promoters, and express vascular endothelial growth factor (VEGF), matrix metalloproteinases (MMPs), and IL-10. Therefore, M2-polarized tumor-associated macrophages (TAMs) can play a vital role in the regulation of tumor migration, invasion, and metastasis in patients [32–34].

**Fig. 1:**
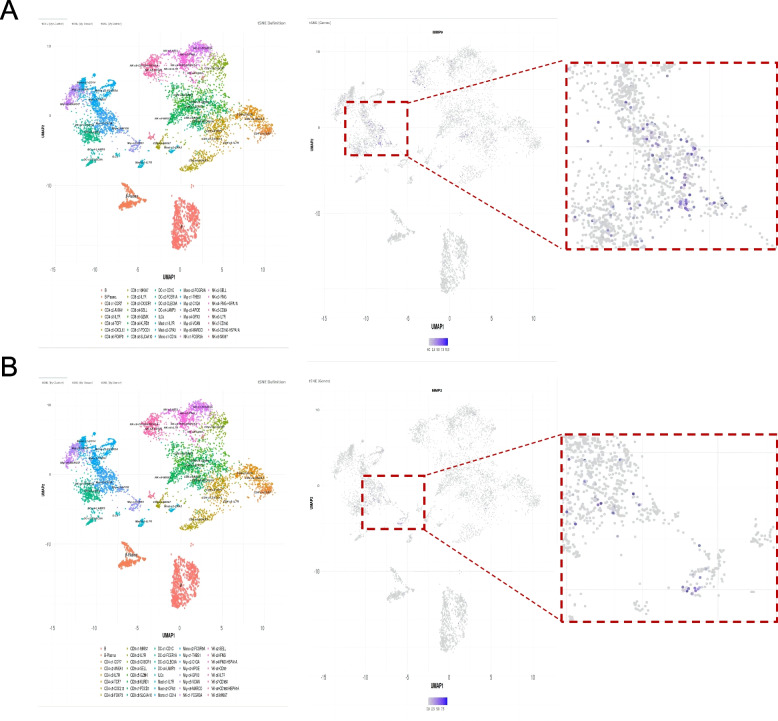
Source of MMP-9 and MMP-2 in the HCC database. **A** MMP-9 was mainly derived from the macrophages. **B** MMP-2 was mainly derived from macrophages

We further searched the HCC database and found that MMP-9 and MMP-2 were primarily produced by the macrophages (Fig. 1). We hypothesized that macrophages destroyed the HCC fibrous capsule by secreting MMP-9 and MMP-2, leading to invasion and metastasis of HCC and a higher recurrence rate in patients post-operation. At present, no studies have reported that HCC macrophage-derived MMP-9 and MMP-2 can be relevant to FC rupture. Therefore, we have conducted this study to investigate the correlation between HCC macrophage-derived MMP-9 and MMP-2 and FC rupture.

## Materials and Methods

### Patients and Tissue Samples

The HCC tissue samples with ruptured and intact FC used in this study were derived from HCC patients (*n* = 45) who had radical hepatectomy at the First Affiliated Hospital of Anhui Medical University, from July 2019 to March 2022. The samples were collected from the ruptured or intact FC area and the adjacent cancerous and paracancerous tissues (0.016–0.20 g). All the patients included in the study were declared as confirmed primary cases through clinical and histological examinations. The samples were stored in a refrigerator at -80 °C and in 4% paraformaldehyde until use. Before the surgery, HCC patients did not receive any kind of chemotherapy, radiotherapy, or targeted therapy for the management of HCC. Table 1 summarizes the clinicopathological data of HCC patients. Each patient was provided informed consent before the surgery and apprized that their samples would be used for biomedical research. This study was approved by the Ethics Committee of the First Affiliated Hospital of Anhui Medical University (PJ-2022–09-19).

**Table 1:** Clinical features of patients (*n* = 45)

**Variables**	**Repured FC (*n* = 26)**		**Intact FC(*n* = 19)**		***P* value**
Age(years), mean ± SD	57.92 ± 12.73		57.32 ± 10.09		0.863
Gender,n(%)					1.000
Male	24	92.31%	17	89.47%	
Female	2	7.69%	2	10.53%	
Hepatitis virus status, n (%)					0.029
HBsAg ( +)	25	96.15%	14	73.68%	
HBsAg (-)	1	3.85%	5	26.32%	
ALT (IU/L), n (%)					0.138
> 40	12	46.15	13	68.42%	
≤ 40	14	53.85%	6	31.58%	
AST (IU/L), n (%)					0.463
> 35	15	57.69%	13	68.42%	
≤ 35	11	42.31%	6	31.58%	
CEA(ng/ml)					0.365
> 5	4	15.38%	5	26.32%	
≤ 5	22	84.62%	14	73.68%	
CA19-9(U/ml)					0.467
> 27	8	30.77%	4	21.05%	
≤ 27	18	69.23%	15	78.95%	
CA125(U/ml)					0.103
> 35	6	23.08%	1	5.26%	
≤ 35	20	76.9%	18	94.74%	
AFP(ng/ml)					0.033
> 400	12	46.15%	3	15.79%	
≤ 400	14	53.85%	16	84.21%	
Vascular invasion, n (%)					0.006
No	15	57.69%	18	94.74%	
Yes	11	42.31%	1	5.26%	
Differentiation,n (%)					0.063
Well or moderately	17	65.38%	17	89.47%	
Poorly	9	34.62%	2	10.53%	
TNM Stage,n (%)					0.021
I	15	57.69%	18	94.74%	
II	10	38.46%	1	5.26%	
III, IV	1	3.85%	0	0%	
Recurrence					0.036
Yes	19	73.08%	8	42.11%	
No	7	26.92%	11	57.89%	

*SD* Stand deviation,;HBsAg( +), hepatitis B surface antigen ( +) means HBV infection, conversely, HBsAg(-) indicates uninfected by HBV, *ALT* Alanine aminotransferase, *AST* Aspartate aminotransferase, *CEA* Carcinoembryonic antigen, *CA19-9* Carbohydrate antigen 19–9, *CA125* Carbohydrate antigen 125, *AFP* Alpha-fetoprotein levels, TNM Stage: *T* Tumor, *N* Nodes, *M* Metastasis, χ2 test

*P* < 0.05

### Hematoxylin–Eosin (H&E) Staining

Ruptured FC tissues (*n* = 3) and intact FC tissues (*n* = 3) were fixed in 4% paraformaldehyde for 24 h, paraffin-embedded, cut into 4-μm-thick sections, and then stained with H&E according to the traditional methods. Each H&E-stained tumor slide was scanned on a Pannoramic MIDI scanner (3DHISTECH Ltd. Budapest, Hungary) and viewed on the software CaseViewer. Two random fields were examined for each section.

### Immunohistochemistry Staining

Immunohistochemical staining of ruptured (*n* = 9) and intact FC tissues (*n* = 6). The rabbit streptavidin–biotin assay system (cat. no. SP-9001; ZSGB-BIO; China) was used for the measure. The 4% paraformaldehyde-fixed, paraffin-embedded tissue was cut at 4 µm thickness, serially sectioned, and the sections were then separated in xylene and rehydrated in a graded ethanol series. After the hydration, antigen retrieve was performed in 20X Tris–EDTA antigen retrieve solution (pH 9.0) (cat. no. G1203; Servicebio; China), Antigen retrieve was performed by boiling in the retrieve solution for 15 min. The endogenous peroxidase was blocked at room temperature for 10 min. The sections were first incubated with normal goat serum for 10–15 min at room temperature and then incubated with the various primary antibodies. These included anti-CD163 antibody (cat. no. ab182422; 1:500; Abcam; UK), anti-MMP-9 antibody (cat. no. 13667; 1:300; CST; USA), anti MMP-2 antibody (cat. no. 40994; 1:150; CST; USA), anti-COL1A1 antibody (cat. no. 72026;1:100; CST; USA), anti-α-SMA antibody (cat. no. 19245;1:600; CST; USA) at 4 °C for overnight. The Biotin-labeled goat anti-rabbit IgG polymer (secondary antibody) (cat. no. SP-9001; ZSGB-BIO; China) was then added for 10–15 min and horseradish enzyme-labeled streptavidin working solution was incubated for 10–15 min. The sections were tested with the Direct Antibody Binding DAB Substrate Kit (cat. no. 8059; 1:150; CST; USA). The films were counterstained with Mayer’s hematoxylin and then sealed. At the end of each step, the sections were washed with phosphate-buffered saline (PBS). Each immunohistochemistry-stained tumor slide was scanned on a Pannoramic MIDI scanner (3DHISTECH Ltd. Budapest, Hungary) and viewed on the software CaseViewer. Two random fields were examined for each section. The expression of each molecule was analyzed by Image J software.

### Immunofluorescence Staining

Paraffin-embedded ruptured FC tissues (*n* = 3) were sliced into 4 μm thick sections, dewaxed, and hydrated with xylene and graded concentration ethanol. After the hydration, antigen retrieve was performed with 20XTris-EDTA antigen retrieve solution (pH 9.0) (cat. no. G1203; Servicebio; China). Antigen retrieve was performed by boiling in the retrieve solution for 10 min. Then, 0.5% TritonX-100 (cat. no. B025; Ebiogo; China) was added dropwise and incubated for 30–60 min in a 37 °C incubator with a cap. Goat serum blocking solution (cat. no. B010; Ebiogo; China) was added dropwise and incubated at 37 °C in an incubator. Thereafter, the double-labeled antibodies (MMP-9 + CD163/MMP-2 + CD163) were mixed at the corresponding dilution ratio (1:300), and the mixed primary antibody was added dropwise and incubated for 60 min in a 37℃ incubator with a cap. Then, immunofluorescent secondary antibodies (goat anti-rabbit IgG (FITC) (cat. no. B029; 1:400; Ebiogo; China), goat anti-mouse IgG (CY3) (cat. no. B026; 1:400; Ebiogo; China) were added dropwise and incubated for 30 min at 37 °C in an incubator with lid protected from light. The tissue slices were sealed with an anti-fluorescence quenching sealer (containing DAPI), (cat. no. B024; Ebiogo; China). Each immunofluorescence-stained tumor slide was scanned on a Pannoramic MIDI scanner (3DHISTECH Ltd. Budapest, Hungary) and viewed on the software CaseViewer.

### Western Blot Analysis

The total proteins from the tissues with the ruptured FC (*n* = 7) (both with cancerous and paracancerous tissues excluded, only the ruptured FC area remained) and its adjacent intact FC (*n* = 7) (both with cancerous and paracancerous tissues excluded, only the intact FC area remained) were extracted on ice, using RIPA lysis buffer (cat. no. P0013B; Beyotime; China) and phosphatase inhibitor (cat. no. P1081; Beyotime; China). The protein concentrations were measured with a BCA protein analysis kit (cat. no. 23225; Thermo Fisher Science; USA), and then proteins were separated by sodium dodecyl sulfate–polyacrylamide gel electrophoresis (SDS-PAGE) and then transferred to nitrocellulose membranes (Bio-Rad; Hercules; CA; USA). The membranes were closed in 5% skim milk for 1 h at room temperature for blocking and then incubated with anti-MMP-9 (cat. no. 13667; 1:300; CST; USA), anti-MMP-2 (cat. no. 40994; 1:150; CST; USA), anti-beta Actin (cat. no. ab8226; 1:10,000; Abcam; UK) specific antibodies were incubated overnight at 4◦ C. After overnight incubation, the membranes were washed 3 times with TBST and then incubated with secondary antibody (cat. no. S0002; 1:10,000; Affinity; China & cat. no. ZB-2301; 1:20,000; ZSGB-BIO; China) for 2 h at the room temperature. Finally, a chemiluminescence analysis was performed. The band quantification of MMP-9 and MMP-2 protein expression between the different groups were analyzed by Image J software and normalized to β-Actin.

### Quantitative Real-Time Polymerase Chain Reaction

Total RNA was isolated from tissue specimens using TRIzol reagent (Invitrogen) according to the manufacturer’s protocol (*n* = 7). After determining the total RNA concentration, reverse transcription to cDNA was carried out using the Evo M-MLV Reverse Transcription Premix Kit (cat. no. AG11728; agbio; China). Quantitative real-time polymerase chain reaction analysis was then performed on a Roche LightCycler 96 using SYBR premix Ex Tap II (Takara). All assays were conducted three times and were performed in triplicate. The data analysis involved the ΔΔCt method. All primers were synthesized by tsingke (Beijing; China). Primers used in this study were as follows:

- MMP-9: Forward primer 5’-GGGACGCAGACATCGTCATC-3’;
- Reverse primer 5’- TCGTCATCGTCGAAATGGGC-3’;
- MMP-2: Forward primer 5’-TGGCAAGTACGGCTTCTGTC-3’;
- Reverse primer 5’- TTCTTGTCGCGGTCGTAGTC -3’;
- β-Actin: Forward primer 5’- CATGTACGTTGCTATCCAGGC-3’;
- Reverse primer5’- CTCCTTAATGTCACGCACGAT -3’.
- β-Actin was considered the endogenous control.

### Enzyme-Linked Immunosorbent Assay (ELISA)

The human MMP-9 kit (cat. no. ml058617-2; mlbio; China) and the human MMP-2 kit (cat. no. ml058669-2; mlbio; China) were used to evaluate the protein concentrations of MMP-9 and MMP-2 in the tissues with ruptured (*n* = 19) and intact (*n* = 19) FC and in serum from ruptured (*n* = 10) and intact (*n* = 10) patients. The tissues were added to the appropriate amount of PBS to make the tissue homogenate, centrifuged at 1000 × g for 10 min, and the supernatant was collected. The blood of HCC patients was collected and then centrifuged at 1000 × g for 10 min and the serum was collected. MMP-9 and MMP-2 protein concentrations were measured by ELISA according to the manufacturer’s instructions. Thereafter, the optical density (OD) value of each well was measured on an ELx800 (Biotek; USA) at a wavelength of 450 nm, A standard curve linear regression equation was then estimated based on standard concentrations and the corresponding OD values. The OD value for each sample was then added to the regression equation to calculate the sample’s concentration. Each sample was analyzed in triplicate.

### Statistical Analysis

Quantitative data were summarized and expressed as mean ± standard error of the mean (SEM). Statistical analysis was conducted using SPSS (version 26.0) and GraphPad Prism (version 8.0) software programs. The student’s t-test and paired t-test were used to analyze the statistical significance between independent groups and paired data, respectively. Chi-square test analysis was used to evaluate the correlation between FC ruptured or not and the clinicopathological parameters of HCC. ROC curve analysis was used to evaluate MMP-9 and MMP-2 for FC rupture diagnostic value. *P* < 0.05 indicated a statistical difference. Each experiment was repeated three times.

## Results

We hypothesized that macrophages destroyed the HCC fibrous capsule by secreting MMP-9 and MMP-2, leading to invasion and metastasis of HCC. In this study, validation by immunohistochemistry and immunofluorescence showed that a significant number of macrophages and their derived MMP-9 and MMP-2 aggregates in the area of the ruptured FC. Next, western blot analysis and Quantitative real-time PCR proved the high expression of MMP-9 and MMP-2 in the area of the ruptured FC. Finally, MMP-9 and MMP-2 were able to discriminate effectively between ruptured FC and intact FC were verified by ELISA. Hence, our finding reveals that macrophage-derived MMP-9 and MMP-2 are closely related to the rupture of the FC of hepatocellular carcinoma leading to tumor invasion.

### FC Rupture is Significantly Correlated with Vascular Invasion, TNM Stage, AFP, and Tumor Recurrence

The different HCC Patients with ruptured FC (*n* = 26) and intact FC (*n* = 19) were included in this study. Overall, ruptured FC patients and intact FC patients were well matched for age and sex (*P* = 0.863 and *P* = 1.000, respectively). Table 1 shows the clinicopathological data of all the patients. We retrospectively analyzed the clinicopathological data of all the patients, using Chi-Squared Test (χ2) to calculate the possible correlation between whether the FC was ruptured or not and the clinicopathological data, and a *P* value < 0.05 was considered statistically significant. There were significant correlations observed between the ruptured FC and vascular invasion (*P* = 0.0006), TNM stage (*P* = 0.021), AFP (ng/ml) (*P* = 0.0033), and recurrence (*P* = 0.036), which could guide the clinical therapy.

### HCC Cells can Invade the Paracancerous Liver Tissue Through the Ruptured Area of FC

**Fig. 2:**
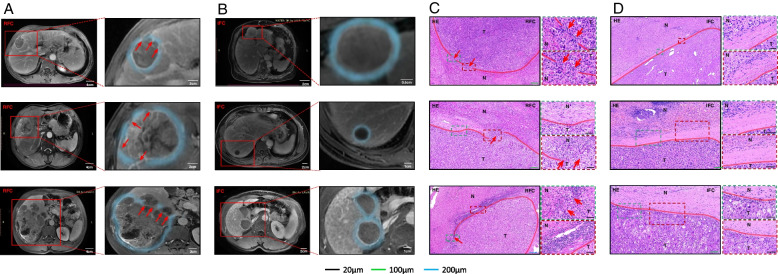
HCC cells can invade the paracancerous liver tissue through the ruptured area of FC. **A** MR image of the ruptured FC (*n* = 3), the blue line is the FC, and the red arrow shows the FC ruptured area whose direction is the direction of the tumor cells through the ruptured FC area to invade the paracancerous liver tissues. **B** MR image of the intact FC (*n* = 3). The blue line is the intact FC. **C** H&E staining of the ruptured FC (*n* = 3), where HCC cells can directly touch the paracancerous liver tissues through the ruptured FC area and then migrate and invade. **D** H&E staining of intact FC (*n* = 3). HCC cells existed within the intact FC due to the restriction of the intact FC but did not have direct contact with the paracancerous liver tissues. HCC tumor tissues are indicated by T and the paracancerous liver tissues are indicated by N. The red line adjacent to the cancerous tissue is the FC area; RFC indicates the ruptured fibrous capsule; IFC indicates the intact fibrous capsule

To validate the route of the tumor invasion to the paracancerous liver tissue, we retrospectively collected Magnetic resonance imaging (MRI) data from HCC patients (*n* = 6), and the results showed that hepatocellular carcinoma cells directly migrated into the paracancerous liver tissues through the ruptured area of FC and then displayed invasion (Fig. 2A). In contrast, HCC cells from intact FC patients were wrapped up in intact FC without contacting the paracancerous liver tissue due to the restriction of FC (Fig. 2B). To further demonstrate our finding, we performed H&E staining on the tissue slides of HCC patients (*n* = 6), and the above phenomenon was noted in their corresponding HE stained slides (Fig. 2C, D). Taken together, these data suggested that FC rupture is a precondition for HCC cell invasion and metastasis.

### Macrophages are Related to FC rupture

**Fig. 3:**
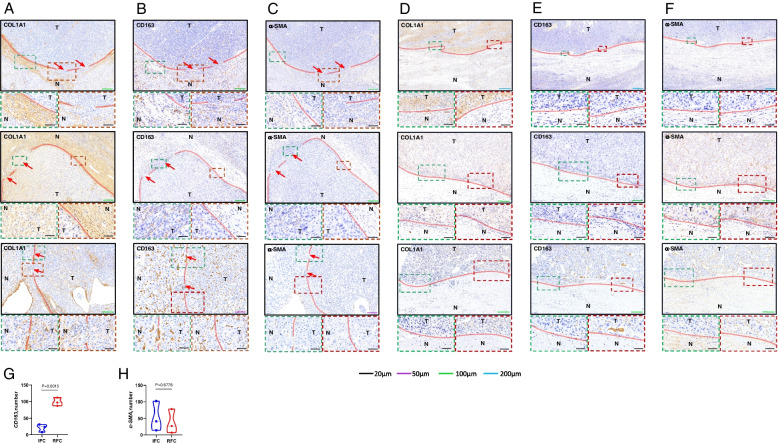
Macrophages are related to FC rupture. **A** Immunohistochemical staining for COL1A1 of the ruptured FC (*n* = 3). **B** Immunohistochemical staining for CD163 of the ruptured FC (*n* = 3). **C** Immunohistochemical staining for α-SMA of the ruptured FC (*n* = 3). **D** Immunohistochemical staining for COL1A1 of the intact FC (*n* = 3). **E** Immunohistochemical staining for CD163 of the intact FC (*n* = 3). **F** Immunohistochemical staining for α-SMA of the intact FC (*n* = 3). **G** CD163 expression in the ruptured FC area was found to be considerably higher than that in the intact FC area (*P* = 0.0015). **H** α-SMA expression did not directly correlate with the presence or lack of FC rupture (*P* = 0.6778). HCC tumor tissues are indicated by T and the paracancerous liver tissues are indicated by N. The red line adjacent to the cancerous tissue is FC; the red arrow indicates the FC ruptured area whose direction is the direction of tumor cells breaking through the ruptured area of FC to invade the paracancerous liver tissue; RFC indicates the ruptured fibrous capsule; IFC indicates the intact fibrous capsule

To verify whether macrophages and HSCs are correlated with FC rupture, we used COL1A1 immunohistochemical staining to identify the location of ruptured and intact FC, and CD163 and α-SMA immunohistochemical staining of the ruptured as well as intact area of FC were performed on all the serial slides (*n* = 6). The results indicated that the ruptured area of FC (Fig. 3A) and the intact area of FC (Fig. 3D) were identified by immunohistochemical staining in COL1A1, ruptured FC had a higher CD163 and α-SMA-positive rate than intact FC (Fig. 3B, C, E, F). It indicated that CD163 and α-SMA expression were associated with FC rupture. To further illustrate that macrophages and HSCs are related to FC rupture, the expression of CD163 and α-SMA were analyzed by Image J software, then statistical analysis was conducted using GraphPad Prism (version 8.0) software programs, which showed that the expression of CD163 in the ruptured area of FC was significantly higher than that in the intact area of FC (*P* = 0.0015) (Fig. 3G), while α-SMA was not correlated (*P* = 0.6778) (Fig. 3H). The above results indicated that the macrophages were massively aggregated in the ruptured FC area and directly associated with FC rupture, whereas activated HSCs expressing α-SMA could not be correlated with FC rupture. To sum up, macrophages are correlated with FC rupture.

### MMP-9 and MMP-2 are Critical Molecules that Lead to FC Rupture

**Fig. 4:**
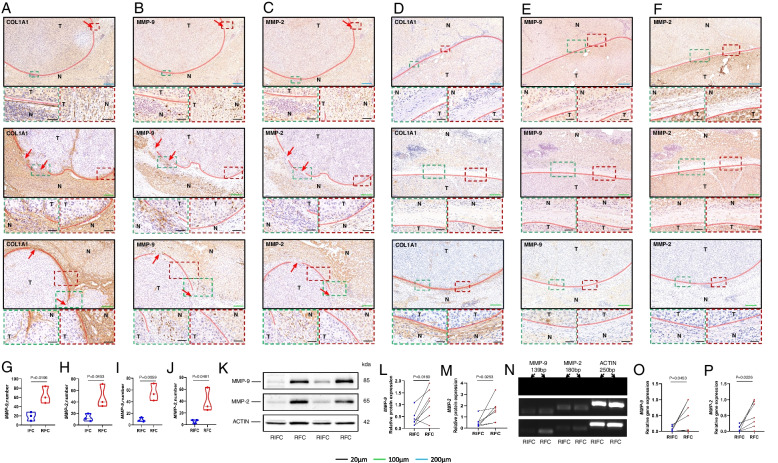
MMP-9 and MMP-2 are critical molecules that lead to FC rupture. **A** Immunohistochemical staining for COL1A1 of the ruptured FC (*n* = 3). **B** Immunohistochemical staining for MMP-9 of the ruptured FC (*n* = 3), where MMP-9 was abundantly aggregated in the ruptured area of FC (*n* = 3). **C** Immunohistochemical staining for MMP-2 of the ruptured FC (*n* = 3), MMP-2 was abundantly aggregated in the ruptured area of FC. **D** Immunohistochemical staining for COL1A1 of the intact FC (*n* = 3). **E** Immunohistochemical staining for MMP-9 of the intact FC (*n* = 3). **F** Immunohistochemical staining for MMP-2 of the intact FC (*n* = 3). **G** The expression of MMP-9 in the ruptured FC area was found to be significantly higher than that in the intact FC area (*P* = 0.0196). **H** MMP-2 expression in the ruptured FC area was observed to be significantly higher than that in the intact FC area (*P* = 0.0453). **I** The expression of MMP-9 in the ruptured FC area was higher in ruptured FC patients than in their RIFC area (*P* = 0.0059). **J** The expression of MMP-2 in the ruptured FC area was higher in the ruptured FC patients than in their RIFC area (*P* = 0.0481). **K** The protein expression of MMP-9 and MMP-2 and ACTIN in the RFC and RIFC areas. **L** MMP-9 protein levels in the RFC area were significantly higher as compared to the RIFC area (*n* = 7) (*P* = 0.0222). **M** MMP-2 protein levels in the RFC area were significantly higher as compared to the RIFC area (*n* = 7) (*P* = 0.0165). **N** The gene expression of MMP-9 and MMP-2 and ACTIN in the RFC and RIFC areas. **O** The mRNA expression level of MMP-9 in the RFC area was significantly higher than that in the RIFC area (*n* = 7) (*P* = 0.0453). **P** The mRNA expression level of MMP-2 in the RFC area was significantly higher than that in the RIFC area (*n* = 7) (*P* = 0.0226). HCC tumor tissues are indicated by T and the paracancerous liver tissues are indicated by N. The red line adjacent to the cancerous tissue is FC; the red arrow indicates the FC ruptured area whose direction is the direction of tumor cells breaking through the ruptured area of FC to invade the paracancerous liver tissue; RFC indicates the ruptured fibrous capsule; IFC indicates the intact fibrous capsule; RIFC indicates the relative intact fibrous capsule

First, we still used COL1A1 immunohistochemical staining, the ruptured area of FC (Fig. 4A) and intact area of FC (Fig. 4D) were demonstrated by COL1A1 immunohistochemical staining. To validate whether MMP-9 and MMP-2 were related to FC rupture, we performed MMP-9 and MMP-2 immunohistochemical staining on all the serial slides. The result indicated that the expression of MMP-9 in the ruptured FC patients was significantly higher than in the intact FC patients (*P* = 0.0196) (Fig. 4B, E) and the expression of MMP-2 in the ruptured FC patients was also significantly higher than in the intact FC patients (*P* = 0.0453) (Fig. 4C, F). To further verify that MMP-9 and MMP-2 are related to FC rupture, the expressions of MMP-9 and MMP-2 were analyzed by Image J software, then statistical analysis was conducted using GraphPad Prism (version 8.0) software programs, the results suggest that MMP-9 and MMP-2 are indeed correlated with FC rupture (Fig. 4G, H). In addition, the expressions of MMP-9 and MMP-2 in the ruptured area of FC were significantly higher compared to that in the relatively intact area of the same ruptured FC patients (*P* = 0.0059/*P* = 0.0481) (Fig. 4I, J).

To further investigate the above views, we further conducted western blot analysis of the ruptured area of FC and its relatively intact area of FC (RIFC) in 7 patients with ruptured FC. The findings indicated that the levels of MMP-9 and MMP-2 proteins in the ruptured area of FC were significantly higher than those in the RIFC (Fig. 4K, L, M). Next, we confirmed these changes in mRNA levels by doing qRT-PCR, the data indicated that MMP-9 and MMP-2 expressions were significantly indeed higher in the FC rupture area than in the RIFC area (Fig. 4N, O, P). In summary, MMP-9 and MMP-2 are related to FC rupture and are the critical molecule that causes FC rupture.

### MMP-9 and MMP-2 are Derived from Macrophages Causing FC Rupture

**Fig. 5:**
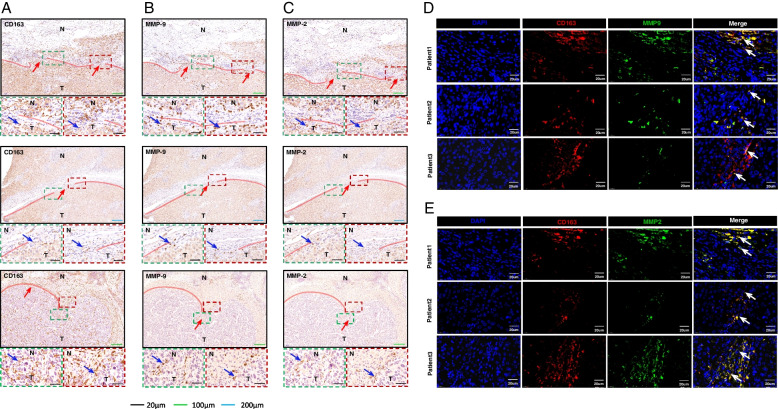
MMP-9 and MMP-2 are derived from macrophages causing FC rupture. **A** Immunohistochemical staining for MMP-9 in the ruptured FC (*n* = 3), and a significant number of the macrophages in the area of the ruptured FC. **B** Immunohistochemical staining for MMP-9 in the ruptured FC (*n* = 3), and a significant number of MMP-9 was aggregated in the ruptured area of the FC. **C** Immunohistochemical staining for MMP-2 in the ruptured FC (*n* = 3), and a significant number of MMP-2 was concentrated in the ruptured area of the FC. **D** Immunofluorescence staining of CD163 and MMP-9 (*n* = 3), with co-localization of CD163 and MMP-9. **E** Immunofluorescence staining of CD163 and MMP-2 (*n* = 3), with co-localization of CD163 and MMP-2. HCC tumor tissues are indicated by T and the paracancerous liver tissues are indicated by N. The red line adjacent to the cancerous tissue is FC; the red arrow indicates the FC ruptured area whose direction is the direction of tumor cells breaking through the ruptured area of FC to invade the paracancerous liver tissue. The blue and white arrows are the points where the three are co-located

To verify whether MMP-9 and MMP-2 are derived from macrophages, we performed immunohistochemical staining and immunofluorescence staining on the ruptured area of FC (all serial slides), we found that CD163 as well as MMP-9 and MMP-2 were both abundantly expressed in the ruptured area of FC in the consecutive slides (Fig. 5A, B, C). Interestingly, in the ruptured area of FC, the area that expressed CD163 was identical to MMP-9 and MMP-2, this is indicative of the colocalization of CD163 as well as MMP-9 and MMP-2. Furthermore, we conducted CD163 as well as MMP-9 and MMP2 co-immunofluorescence staining to validate this observation. The results indicated that there were substantial co-localizations of CD163 as well as MMP-9 and MMP-2 (Fig. 5D, E) in the ruptured area of FC. Based on the above results, it was concluded that a significant number of macrophages aggregated in the ruptured area of FC and their derived MMP-9 and MMP-2 are correlated with FC rupture as well as tumor invasion.

### Clinical Diagnostic Value of MMP-9 and MMP-2 for FC Rupture

**Fig. 6:**
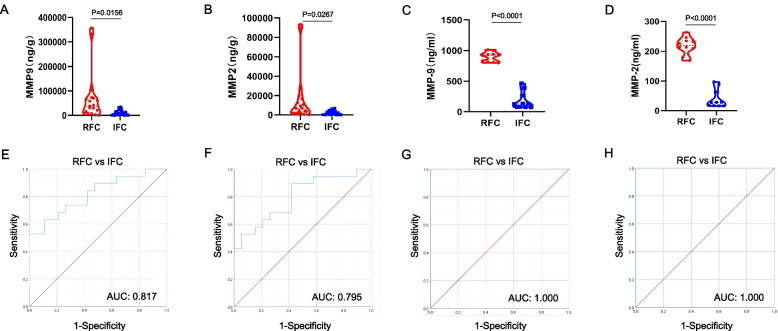
Clinical diagnostic value of MMP-9 and MMP-2 for FC rupture. **A** The protein concentration of MMP-9 in the ruptured FC tissues was found to be significantly higher than that in intact FC tissues (*P* = 0.0156). **B** The protein concentration of MMP-2 in ruptured FC tissues was observed significantly higher than that in intact FC tissues (*P* = 0.0267). **C** The protein concentration of MMP-9 in serum was significantly higher in ruptured FC patients than in intact FC patients (*p* < 0.0001). **D** The protein concentration of MMP-2 in serum was significantly higher in ruptured FC patients than in intact FC patients (*p* < 0.0001). **E** ROC curve to determine the optimal cutoff value for tissue MMP-9. The optimal cutoff value for MMP-9 was 27,007.56 ng/g (sensitivity 63.2%; specificity 89.5%). **F** ROC curve to determine the optimal cutoff value for tissue MMP-2. The optimal cutoff value for MMP-2 was 1622.70 ng/g (sensitivity 89.5%; specificity 57.9%). **G** ROC curve to determine the optimal cutoff value for serum MMP-9. The optimal cutoff value for MMP-9 was 630.8191 ng/ml (sensitivity 100%; specificity 100%). **H** ROC curve to determine the optimal cutoff value for serum MMP-2. The optimal cutoff value for MMP-2 was 132.9021 ng/ml (sensitivity 100%; specificity 100%). RFC indicates the ruptured fibrous capsule; IFC indicates the intact fibrous capsule

To determine the potential diagnostic value of MMP-9 and MMP-2 for FC rupture or not, we measured protein concentrations in ruptured tissue (*n* = 19) and intact FC tissue (*n* = 19) and in serum from patients with ruptured FC (*n* = 10) and intact FC (*n* = 10) by ELISA. All ELISA data was then analyzed with Prism software 8.0 (GraphPad Software) and cutoff values were calculated with SPSS 26.0 (IBM; USA). The results demonstrated that the expression of MMP-9 in the ruptured FC tissues was significantly higher compared to intact FC tissues (*P* = 0.0156) (Fig. 6A), the expression of MMP-2 in the ruptured FC tissues was significantly higher than intact FC tissues (*P* = 0.0267) (Fig. 6B), the expression of MMP-9 in the serum of patients with ruptured FC was significantly higher than that of patients with intact FC (*P* < 0.0001) (Fig. 6C), and the expression of MMP-2 in the serum of patients with ruptured FC was significantly higher than that of patients with intact FC (*P* < 0.0001) (Fig. 6D). This further validated the view that MMP-9 and MMP-2 are correlated with FC rupture.

Furthermore, the ROC curve was used to determine the diagnostic value of MMP-9 and MMP-2 for FC rupture, depending on the MMP-9 and MMP-2 protein concentrations in tissue and serum. The optimal cutoff value for MMP-9 in tissue was 27,007.56 ng/g, with a sensitivity of 63.2% and specificity of 89.5%, and MMP-9 showed an AUC of 0.817 for discriminating ruptured FC from intact FC (Fig. 6E), and the optimal cutoff value for MMP-2 in tissue was 1622.70 ng/g, with a sensitivity of 89.5% and specificity of 57.9%, and MMP-2 showed an AUC of 0.795 for discriminating ruptured FC from intact FC (Fig. 6F). The optimal cutoff value for MMP-9 in serum was 630.8191 ng/ml, with a sensitivity of 100.0% and specificity of 100.0%, and MMP-9 showed an AUC of 1.000 for discriminating ruptured FC from intact FC (Fig. 6G), and the optimal cutoff value for MMP-2 in serum was 132.9021 ng/ml, with a sensitivity of 100.0% and specificity of 100.0%, and MMP-2 showed an AUC of 1.000 for discriminating ruptured FC from intact FC (Fig. 6H).

In conclusion, MMP-9 and MMP-2 were able to effectively differentiate between ruptured FC and intact FC, both on the tissue level and on the serum level, suggesting that it has great potential in diagnosing whether FC is ruptured in patients with hepatocellular carcinoma. Serum MMP-9 and MMP-2 cutoff values can effectively predict whether FC is ruptured in patients with hepatocellular carcinoma pre-operatively, determine the range of surgery, and provide a new diagnostic method for clinical therapy, which is of guiding significance. And tissue MMP-9 and MMP-2 cutoff values can further determine whether FC is ruptured post-operatively, thereby predicting patients’ post-operative prognosis and recurrence.

## Discussion

HCC is one of the most common malignancies worldwide and the tumor is prone to recurrence after surgery and remains a high mortality rate. Therefore, it is critical to investigate the causes of HCC recurrence and metastasis. A number of previous studies have reported that the presence of intact FC surrounding HCC is essential to limit the migration and invasion of tumor cells, and FC primarily consists of type I collagen [16, 17]. In the present study, we performed COL1A1 immunohistochemical staining on the patient’s tissue slides, which also further confirmed this view. The results indicated that the FC was composed of type I collagen in patients with ruptured FC that invaded the paracancerous hepatic tissue through the ruptured area of FC. On the contrary, HCC cells in patients with intact FC existed within the FC due to the barrier of intact FC, which was similar to the results of the existing study [20].

The rupture of FC is a very important clinical phenomenon in HCC invasion and metastasis. Vihinen et al. found that MMP-9 and MMP-2 were believed to be crucial in the invasion of malignant tumors [27]. Chen et al. revealed that MMP-9 and MMP-2 were critical for tumor cell invasion and metastasis [35]. Huang et al. reported that MMP-9 and MMP-2 expression and activity had been shown to play a key role in many human cancers with metastatic capability [23]. Zhang et al. revealed that MMP-9 and MMP-2 promoted HCC invasion and significant correlation with poor survival [10]. Chen et al. identified that MMP-9 and MMP-2 were closely associated with invasion and metastasis in HCC [36]. Li et al. revealed that MMP-9 and MMP-2 were considered to be involved in tumor metastasis [37]. However, no previous study has investigated the correlation between MMP-9 and MMP-2 and FC rupture in hepatocellular carcinoma.

In this study, our results indicated that the rupture area of FC aggregated a significant number of macrophages and that MMP-9 and MMP-2 secreted by macrophages can rupture FC composed of type I collagen with significantly higher expression than intact FC area. Further verification was carried out via western blot and qRT-PCR, and the obtained results revealed that MMP-9 and MMP-2 showed considerable differential expression in ruptured and intact FC, thus confirming that FC rupture significantly correlated with macrophage-derived MMP-9 and MMP-2. Moreover, MMP-9 and MMP-2 were able to discriminate effectively from ruptured FC and intact FC and hence determine the range of surgery preoperatively in HCC patients as well as predict the likelihood of recurrence and metastasis in HCC patients. Therefore, it is suggested that when performing surgical resection, it is necessary to expand the range of tumor resection for patients with ruptured FC identified by the levels of serum MMP-9 and MMP-2.

Further research still needs to be performed in the future. First, the number of validated samples was relatively insufficient, and more samples will be required for further confirmation. Second, the extensive molecular mechanism of MMP-9 and MMP-2 in HCC fibrous capsules has not been explored. Third, novel strategies inhibit the expressions of MMP-9 and MMP-2 and thus reduce HCC invasion, which can result in lower TNM stage in patients and lower tumor metastasis recurrence rate. In a future study, we will further explore these questions.

## Conclusion

In summary, our study provides a novel perspective on the potential role of MMP-9 and MMP-2 to promote invasion and metastasis of HCC and reveals that patients with ruptured FC aggregated a significant number of macrophages and macrophage-derived MMP-9 and MMP-2 in the ruptured FC area, which could destroy the FC and caused HCC cells to invade from the tumor area to the paracancerous liver tissues through the ruptured FC area. In addition, MMP-9 and MMP-2 are able to discriminate effectively between ruptured FC and intact FC and can effectively guide the clinical to identify the ruptured FC and hence reduce HCC recurrence and metastasis.

## Data Availability

All datasets and materials involved in this study are provided in the article. Publicly available datasets were analyzed in this study. This data can be found in The HCC database (http://cancer-pku.cn:3838/HCC/).
